# Metformin Use and Lung Cancer Risk in Diabetic Patients: A Systematic Review and Meta-Analysis

**DOI:** 10.1155/2019/6230162

**Published:** 2019-02-10

**Authors:** Long Yao, Mengke Liu, Yunlong Huang, Kaiming Wu, Xin Huang, Yuan Zhao, Wei He, Renquan Zhang

**Affiliations:** ^1^Department of Thoracic Surgery, The First Affiliated Hospital of Anhui Medical University, 218# Ji Xi Road, Hefei, 230000 Anhui, China; ^2^The First Clinical Medical College, Anhui Medical University, 81# Mei Shan Road, Hefei, 230032 Anhui, China; ^3^School of Basic Medical Science, Anhui Medical University, 81# Mei Shan Road, Hefei, 230032 Anhui, China

## Abstract

**Background:**

Antidiabetic medications (ADMs) can alter the risk of different types of cancer, but the relationship between lung cancer incidence and metformin remains controversial. Our aim was to quantitatively estimate the relationship between incidences of lung cancer and metformin in patients with diabetes in this meta-analysis.

**Methods:**

We performed a search in PubMed, Embase, ISI Web of Science, and Cochrane Library until September 20, 2017. The odds ratio (OR), relative risk (RR) or hazard ratio (HR), and 95% confidence interval (95% CI) were estimated using the random-effect model. The Newcastle-Ottawa Scale (NOS) was used to assess the study quality.

**Results:**

A total of 13 studies (10 cohort studies and 3 case-control studies) were included in the meta-analysis. Compared to nonmetformin users, metformin probably decreased lung cancer incidence in diabetic patients (RR = 0.89; 95% CI, 0.83-0.96; *P* = 0.002) with significant heterogeneity (*Q* = 35.47, *I* ^2^ = 66%, *P* = 0.0004). Subgroup analysis showed that cohort studies (RR = 0.91; 95% CI, 0.85-0.98; *P* = 0.008), location in Europe (RR = 0.90; 95% CI, 0.86-0.94; *P* < 0.0001), the control drug of the sulfonylurea group (RR = 0.91; 95% CI, 0.86-0.96; *P* = 0.001), and adjusting for smoking (RR = 0.86; 95% CI, 0.75-1.00; *P* = 0.05) may be related to lower lung cancer risk. No significant publication bias was detected using a funnel plot.

**Conclusion:**

Metformin use was related to a lower lung cancer risk in diabetic patients compared to nonusers, but this result was retrieved from observational studies and our findings need more well-designed RCTs to confirm the association.

## 1. Introduction

Lung cancer is one of the leading causes of cancer-related deaths in the world [1], and despite improvements in early prevention and treatment, the etiologic mechanism is still unclear. A number of research studies have reported several risk factors, of which smoking is the most important one. Other risk factors include the low intake of vegetables and fruits, exposure to asbestos or other carcinogens, previous lung diseases, and lung cancer history [2–4]. Once lung cancer is diagnosed, it is typically in an advanced stage, which leads to a bad prognosis, with a reported five-year survival rate of approximately 15% [5]. Therefore, prevention of lung cancer should be given utmost attention.

Diabetes mellitus (DM) is a worldwide metabolic disease, which is reported to increase the risk of a variety of cancers, such as lung, liver, kidney, and breast cancer [6–9]. DM is characterized as chronic hyperglycemia and abnormal carbohydrate, fat, and protein metabolism. Several epidemiologic studies have demonstrated that antidiabetic medications (ADMs) may increase or reduce cancer risk [10, 11]. Metformin is an oral antidiabetic medication, ubiquitously used for treating diabetic patients. Metformin exerts antineoplastic effects by the following two mechanisms: (i) activating adenosine monophosphate-activated protein kinase (AMPK) and (ii) inhibiting protein synthesis [12].

Studies evaluating the link between lung cancer risk and metformin use among patients with diabetes have produced inconsistent results. Lai et al. [13] found a decline in the lung cancer incidence in diabetic patients associated with the usage of metformin; this was in accordance with the research by Mazzone et al. [14] and Hsieh et al. [15]. However, Smiechowski et al. [16] and Sakoda et al. [17] found that metformin was not related to decreased lung cancer incidence. And meta-analyses also demonstrated controversial results. Wu et al. [18] pooled 15 observational studies and concluded that metformin was associated with a 15% reduction in lung cancer risk, and by including 15 studies, Zhang et al. [19] concluded from 6 studies that metformin therapy was associated with estimated reductions of 29% in lung cancer and 15% in cancer of the respiratory system. Nie et al. [20] concluded that there is no significant association between the incidence of lung cancer and metformin. In addition, we found that all of these meta-analyses included a few studies with low sample sizes or overlapping data were used, and therefore, the results may not be generalizable. For example, Zhang et al. [19] only included four studies analyzing the relationship between metformin and lung cancer risks. Hence, to better understand the relationship and evaluate the quality of studies, we performed this meta-analysis to investigate the relationship between metformin use and the incidence of lung cancer in diabetic patients.

## 2. Materials and Methods

### 2.1. Literature Search

We conducted a study to investigate the association between the incidence of lung cancer and metformin or biguanides in diabetic patients. Databases including PubMed, Embase, ISI Web of Science, and Cochrane Library were searched until September 20, 2017. We used the following keywords or MeSH terms: (Metformin or biguanides) and (cancer or carcinoma or neoplasm or tumor) and lung. The reference lists of relevant reviews and meta-analyses were manually checked for other potential articles that may have been missed in the initial search.

### 2.2. Selection Criteria

The inclusion criteria were as follows: (a) cohort or case-control studies, (b) assesses the relationship between incidence of lung cancer and metformin in patients with diabetes, and (c) odds ratio (OR), relative risk (RR) or hazard ratio (HR), and 95% confidence interval (95% CI) were reported (or adequate data existed to calculate these values). When several publications came from the same population, the most recent or comprehensive one was included. The comparators were defined as any treatment not including metformin.

### 2.3. Data Extraction and Quality Assessment

Data were extracted by two reviewers independently from all included publications on a standardized form. The following data were extracted from the selected studies: first author, publication year, country, study design, study time or follow-up time, case source, comparison, and unadjusted and/or adjusted ratio (HR/OR/RR and their 95% confidence intervals (95% CIs)), which were defined as a tool to measure associations. Any disagreements in the extraction were resolved by discussion when we refer to the original articles. The quality of the included studies was evaluated by the Newcastle-Ottawa Scale (NOS), which is a recommendation by the Cochrane Non-Randomized Studies Methods Working Group [20].

### 2.4. Statistical Analysis

The RRs from the cohort studies or ORs from the case-control studies were adopted to assess the estimated risk between metformin and lung cancer in the present study. Based on inverse variance, studies were pooled and measured using the DerSimonian and Laird statistic in the random-effect model, which considered both within-study and between-study variations. *P* < 0.05 and the value one were not included in the 95% CI, indicating a statistically significant difference among all studies. Cochran's *Q* test and *I* ^2^ statistics were used to evaluate heterogeneity among the studies. For the *Q* statistic, a *P* value less than 0.10 was considered as having statistically significant heterogeneity, and for *I* ^2^, a value more than 50% was considered to be having severe heterogeneity. Then, subgroup analyses were conducted by study design and geographic area and were adjusted for smoking, BMI, HbA1C, alcohol use, and other glucose-lowering drugs. A sensitivity analysis was conducted using the following methodology: exclude each study and analyze the rest. Finally, the funnel plot for asymmetry was carried out to detect publication bias. All analyses were performed with RevMan version 5.3.

## 3. Results

### 3.1. Search Results

**Figure 1:**
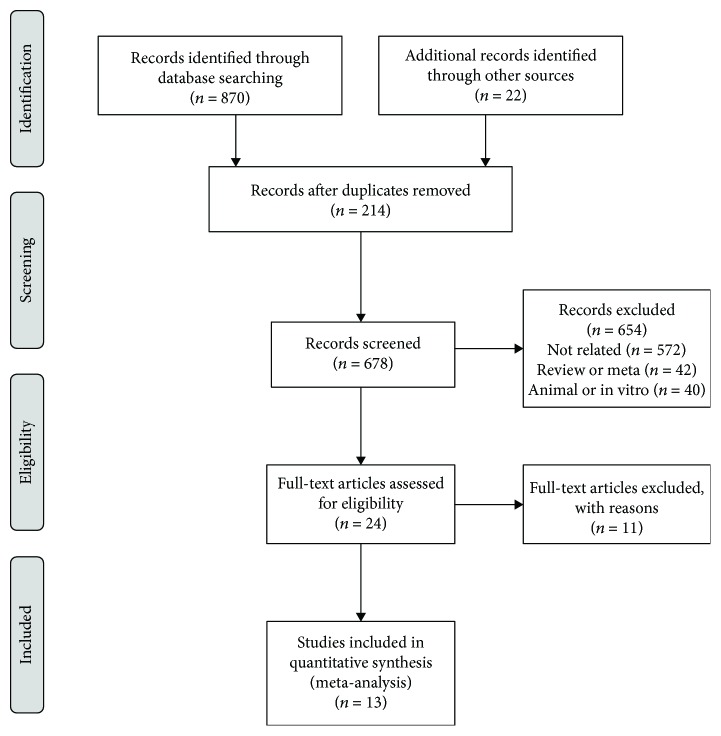
Flow chart of study selection.

A total of 892 studies were obtained using the above search strategy, and 17 studies were identified through initial research. Of these studies, Hall et al. [21], Bodmer et al. [22], and Smiechowski et al. [16] assessed the same population from the U.K. General Practice Research database, and the populations in Ferrara et al.'s study [23] and Sakoda et al.'s study [17] were both from the Kaiser Permanente Northern California (KPNC) Diabetes Registry. After excluding these articles, 13 studies fulfilled the inclusion criteria and were pooled in our meta-analysis [13–17, 24–31] (Figure 1).

### 3.2. Characteristics of the Included Studies

The main characteristics of the 13 studies, including year, first author, country, study design, time period, case source, comparison, and unadjusted and/or unadjusted estimated effect with 95% CI are shown in Table 1. Three studies were case-control studies, and ten studies were cohort studies. Most studies included men and women, except for one study (Luo et al. [27]) that consisted of women only. Four studies were from Asia, five were from Europe, and the remaining four were from North America. Six studies were hospital-based, and the remaining were population-based. The control group in eight studies was diabetic patients with no glucose-lowering drugs, while the others were sulfonylurea or insulin. The estimated risk between lung cancer and metformin in all studies was acquired by adjusting several confounders (Table 2); among them, eight studies were adjusted for BMI, six studies adjusted for smoking, four studies adjusted for HbA1C, five studies adjusted for alcohol consumption, and four studies adjusted for glucose-lowing drugs.

### 3.3. Analysis

**Figure 2:**
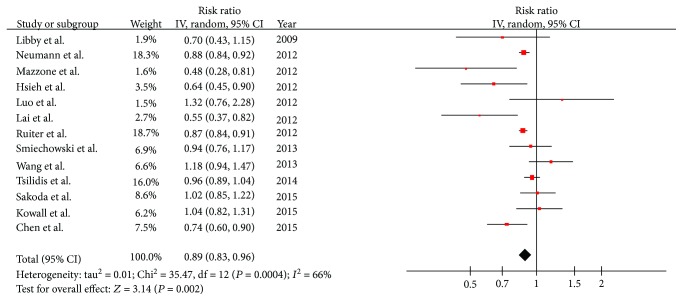
Random-effect meta-analysis of the association between metformin and lung cancer.

The pooled RR from 13 studies is shown in Figure 2. On meta-analysis of all 13 included studies assessing the relationship between the risk of lung cancer and metformin use in diabetic patients, it is shown that the use of metformin was related to a statistically significant 11% reduction in lung cancer incidence (RR = 0.89; 95% CI, 0.83-0.96; *P* = 0.002). Significant heterogeneity was found among these studies (*Q* = 35.47, *I* ^2^ = 66%, *P* = 0.0004).

To confirm the stability and validity of the overall results, we performed a sensitivity analysis by sequentially excluding each study and analyzing the rest. These analyses verified the stability of the result of the significant relationship between the incidence of lung cancer and metformin in diabetic patients. For instance, when we excluded the study with the highest weight, the pooled RR remained significant (RR = 0.89; 95% CI, 0.81-0.98; *P* = 0.01) but still with significant heterogeneity (*I* ^2^ = 68%, *P* = 0.0003).

Then, subgroup analysis was conducted by different characteristics (Table 3) in order to find potential sources of heterogeneity in these studies and test the effect of the study characteristics on the overall result. In the analysis stratified by study design, no vital association was found between the risk of lung cancer and metformin use in diabetic patients (RR = 0.70; 95% CI, 0.47-1.03; *P* = 0.07) in case-control studies, while in cohort studies, a significant association was found (RR = 0.91; 95% CI, 0.85-0.98; *P* = 0.008). By study location, Asia (RR = 0.76; 95% CI, 0.55-1.06; *P* = 0.1) and North America (RR = 0.92; 95% CI, 0.71-1.19; *P* = 0.51) showed no significant association between the incidence of lung cancer and metformin use in diabetic patients, with high heterogeneity (*Q* = 16.89, *I* ^2^ = 82%, *P* = 0.0007 and *Q* = 8.43, *I* ^2^ = 64%, *P* = 0.04, respectively). In contrast, a significant association was found in European (RR = 0.90; 95% CI, 0.86-0.94; *P* < 0.0001) studies with little heterogeneity (*Q* = 7.33, *I* ^2^ = 45%, *P* = 0.12). In the analysis stratified by control group drugs, a no-drug or insulin study had a null relationship between the incidence of lung cancer and metformin in diabetic patients (RR = 0.89; 95% CI, 0.77-1.03; *P* = 0.13 and RR = 0.97; 95% CI, 0.75-1.26; *P* = 0.84, respectively). In contrast, the sulfonylurea group was considered to have a significant association (RR = 0.91; 95% CI, 0.86-0.96; *P* = 0.001). In addition, the sulfonylurea and insulin groups had little heterogeneity (*Q* = 5.09, *I* ^2^ = 21%, *P* = 0.28 and *Q* = 0.06, *I* ^2^ = 0%, *P* = 0.97, respectively).

**Figure 3:**
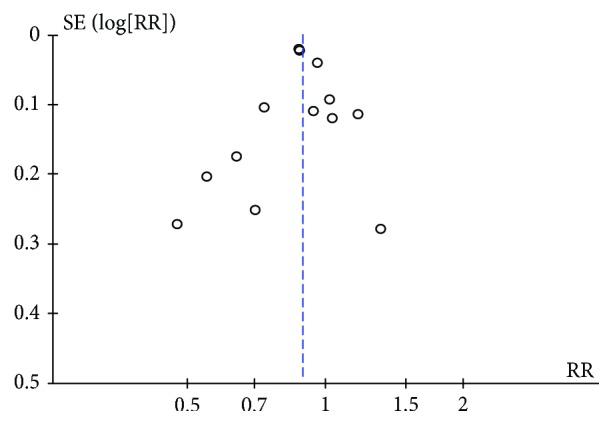
Publication bias detected by funnel plot.

Several pivotal confounders were also investigated, including BMI or obesity, smoking, HbA1C, alcohol consumption, and other glucose-lowing drugs. When the analysis was stratified by adjusting for BMI or obesity, HbA1C, and alcohol consumption, no significant association was found among these subgroups. As regards smoking, an estimated 14% reduction was found between metformin use and lung cancer risks (RR = 0.86; 95% CI, 0.75-1.00; *P* = 0.05), with high heterogeneity (*Q* = 14.11, *I* ^2^ = 65%, *P* = 0.01). With other glucose-lowering drugs, a crucial association was found between lung cancer incidence and metformin use in patients with diabetes (RR = 0.90; 95% CI, 0.84-0.97; *P* = 0.004), with little heterogeneity (*Q* = 3.51, *I* ^2^ = 15%, *P* = 0.32). The publication bias was assessed using a funnel plot, and no significant publication bias was detected as there is no visually apparent asymmetry (Figure 3).

## 4. Discussion

In this meta-analysis assessing the relationship between the risk of lung cancer and metformin use among diabetic patients, we found that metformin was strongly related to decreased lung cancer risk (RR = 0.89; 95% CI, 0.83-0.96; *P* = 0.002). We also found that in 2 RCTs, namely, ADOPT and RECORD [32, 33], this protective effect disappeared (OR = 0.65; 95% CI, 0.33-1.26); however, as most of our studies were observational and there were only two RCTs, we did not include RCTs in our meta-analysis. Our result is consistent with some previous meta-analyses, such as Zhang et al. [19] and Wu et al. [18], while more studies were included in our analysis making our result more persuasive and stable. Subgroup analysis showed that sulfonylurea as control drugs and smoking status were associated with lower lung cancer risks, which suggested that smoking and antidiabetic medication would lead to overestimation of the protective effect of metformin on risk of lung cancer. Smoking is one of the most important risk factors leading to lung cancer verified by plenty of research. However, it is still an unknown problem among smokers and nonsmokers whether metformin will exert different chemopreventive effects on lung cancer. In one animal experiment, Izzotti et al. [34] reported that oral metformin is able to protect the mouse lung from MCS- (mainstream cigarette smoking-) induced DNA damage and to modulate plenty of miRNAs that participated in pulmonary carcinogenesis. More studies are required to investigate, compared to nonsmokers, whether smokers benefit more from metformin treatment. Considerable heterogeneity was found across the included studies that could not be offset by study design, case source, or location. However, little heterogeneity was seen with insulin or sulfonylurea as control drugs. In addition, a subgroup analysis of the studies adjusting for other glucose-lowering drugs showed little heterogeneity; therefore, the existing heterogeneity may be from glucose-lowering drugs. From the sensitivity analysis and funnel plot, our analysis seemed to be stable.

Metformin is an oral antidiabetic drug regarded as the first choice for the treatment of diabetes mellitus (DM), which has been reported to have a potential anticancer role in some solid tumors, such as lung, hepatocellular, and melanoma [35–37]. In NSCLC (non-small-cell lung cancer) patients, metformin combined with tyrosine kinase inhibitors (TKIs) could harbor mutations in EGFR (epidermal growth factor receptor) to reduce resistance to TKI and prolong the overall survival of patients [38]. Metformin combined with gefitinib produced a synergistic effect in LKB1 wild-type NSCLC cells [39], and metformin has the ability to inhibit the metastasis of Lewis lung cancer [40] through the adenosine monophosphate-activated protein kinase *α*1 pathway. Metformin and cisplatin are synergistic in the NCI-H460 cell line [41], and combination of two drugs was more effective than the use of cisplatin as a monotherapy. Several prospective trials are ongoing to assess the effect of metformin in combination with standard chemotherapy in NSCLC patients [42, 43]. Therefore, along with other chemotherapy drugs, metformin may be used in lung cancer patients.

There are several strengths in the present meta-analysis. First, a large number of studies were included and all of the studies were observational, which may make the results more reliable. Second, the studies were selected by rigorous criteria. Hall et al. [21], Bodmer et al. [22], and Smiechowski et al. [16] had the same population from the U.K. General Practice Research database, Ferrara et al. [23] and Sakoda et al. [17] were both from the Kaiser Permanente Northern California (KPNC) Diabetes Registry, and several meta-analyses included these studies of overlapping populations at the same time, which could affect the result; therefore, we only included the most recent and comprehensive studies. Third, all included studies, based on NOS, were of high or moderate quality.

Nevertheless, our meta-analysis has some limitations. First, based on the inclusion criteria, all of the included studies were observational studies; therefore, the association observed between lung cancer incidence and metformin is affected by confounding factors. Inadequate vital-confounder adjustments might lead to spurious relationships between metformin and lung cancer risk. Smoking and previous lung diseases were strongly related to the incidence of lung cancer, and obesity is reported to decrease lung cancer risk, while no studies adjusted for these factors simultaneously. Other confounders, such as vitamin intake, drinking, and physical activity may affect the results as well. Second, the hyperglycemia severity or levels of glycated hemoglobin were not reported in these included studies, so we could not further assess the relationship between hyperglycemic severity and the risk of lung cancer. Third, the included studies did not differentiate between type 1 and type 2 diabetes, and the two types of diabetes may have different associations. Likewise, the studies did not provide lung cancer types, so we were unable to perform subgroup analyses based on each type of lung cancer. Last but not the least, most of observational studies were based on health care databases, resulting in an important amount of immortal time that was either misclassified or excluded. This immortal time bias led to an overestimation of the benefits of medical therapy. Therefore, possible overestimation of the effect requires more well-designed observational studies or randomized controlled trials on association between metformin and lung cancer.

To conclude, metformin use appeared to decrease the incidence of lung cancer in diabetic patients according to the present meta-analysis. Due to significant heterogeneity and possible bias, more investigations, especially well-designed randomized controlled trials, are needed to validate the relationship.

## Figures and Tables

**Table 1:** Characteristics of the included studies.

First author	Year	Country	Study design	Time period	Case source	Comparison	Estimates and 95% CI		Study quality
							Unadjusted	Adjusted	
Libby	2009	UK	Cohort (PB)	1994-2003	The resident population of Tayside Health Board	Metformin vs. nonmetformin	0.49 (0.32-0.74)	0.70 (0.43-1.15)	8
Lai	2012	Taiwan, China	Cohort (PB)	2000-2008	National Health Research Institutes in Taiwan	Metformin vs. nonmetformin	0.43 (0.29-0.63)	0.55 (0.37-0.82)	7
Ruiter	2012	Netherlands	Cohort (HB)	1998-2008	PHARMO Record Linkage System	Metformin vs. Sul	NA	0.87 (0.84-0.91)	7
Neumann	2012	France	Cohort (PB)	2006-2009	French national health insurance information	Metformin vs. nonmetformin	NA	0.88 (0.84-0.92)	7
Mazzone	2012	USA	Case-control (HB)	2001-2011	Cleveland Clinic Health System	Metformin vs. nonmetformin	NA	0.48 (0.28-0.81)	6
Hsieh	2012	Taiwan, China	Case-control (PB)	2000-2008	Taiwan's National Health Insurance Medical Claims Database	Metformin vs. Sul Metformin vs. Ins	NA	0.64 (0.45-0.9) 0.95 (0.46-1.95)	7
							NA		
Luo	2012	USA	Cohort (HB)	1993-2010	Women's Health Initiative (WHI) study	Metformin vs. nonmetformin	NA	1.32 (0.76-2.28)	8
Smiechowski	2013	Canada	Case-control (PB)	1988-2009	U.K. General Practice Research database	Metformin vs. nonmetformin	0.97 (NA)	0.94 (0.76-1.17)	8
Wang	2013	Taiwan, China	Cohort (PB)	1998-2009	National Health Insurance datasets	Metformin vs. nonmetformin	NA	1.11 (0.94-1.47)	7
Tsilidis	2014	UK	Cohort (HB)	1987-2010	U.K. Clinical Practice Research Datalink	Metformin vs. Sul	NA	0.96 (0.89-1.04)	8
Sakoda	2015	USA	Cohort (PB)	1997-2012	KPNC Diabetes Registry	Metformin vs. nonmetformin	NA	1.02 (0.85-1.22)	8
Kowall	2015	Germany and the UK	Cohort (HB)	1995-2013	Disease Analyzer database	Metformin vs. Sul Metformin vs. Ins	0.81 (0.65-1.01)	1.04 (0.82-1.31)	7
							1.28 (0.81-2.04)	1.03 (0.64-1.64)	
Chen	2015	Taiwan, China	Cohort (HB)	1998-2008	Longitudinal Health Insurance Dataset	Metformin vs. Sul Metformin vs. Ins	0.72 (0.58-0.88)	0.74 (0.60-0.90)	8
							0.85 (0.61-1.18)	0.95 (0.68-1.35)	

Abbreviations: PB: population-based; HB: hospital-based; NA: not available; KPNC: the Kaiser Permanente Northern California Diabetes Registry; Sul: sulfonylurea; Ins: insulin.

**Table 2:** Adjustment variables of the included studies.

Study	Adjustment
Libby et al.	Age, sex, smoking, deprivation, BMI, HbA1C, insulin use, and sulfonylurea use
Lai et al.	Sex, age, pulmonary tuberculosis, chronic obstructive pulmonary disease, and propensity score
Ruiter et al.	Age at first OGLD prescription, sex, year in which the first OGLD prescription was dispensed, number of unique drugs used in the year, and number of hospitalizations in the year before the start of the OGLD
Neumann et al.	Age, sex (when applicable), and exposure to glucose-lowering drugs
Mazzone et al.	Medication use, BMI, HbA1C, and pack-years of smoking
Hsieh et al.	Sex and age
Luo et al.	Age, ethnicity, education, BMI, waist-to-hip ratio, recreational physical activity, alcohol intake, total energy intake, percent calories from fat, total fruit intake, total vegetable intake, history of hormone therapy use, and different treatment assignments for clinical trials
Smiechowski et al.	Diabetes duration, HbA1c, obesity, smoking, excessive alcohol use, previous cancer, chronic obstructive pulmonary disease, asthma, nonsteroidal anti-inflammatory drugs, aspirin, statins, and other antidiabetic drugs
Wang et al.	Age, sex, and occupation
Tsilidis et al.	Smoking status, BMI, alcohol consumption, use of aspirin or NSAIDs and statins, diabetes duration, and year of first antidiabetes prescription
Sakoda et al.	Gender, race/ethnicity, birth year, diabetes duration, BMI, alcohol use, Charlson comorbidity index, and other diabetes medications
Kowall et al.	Age at first diabetes medication, sex, country (the UK or Germany), time between first diagnosis of diabetes and prescription of first diabetes drug, obesity, hypertension, hyperlipidemia, prevalence of microcomplications (retinopathy, neuropathy, or nephropathy), Charlson index, use of antihypertensives, use of antithrombotic agents, use of aspirin, use of statins, use of nonsteroidal anti-inflammatory drugs, and use of contraceptives
Chen et al.	Age, sex, Charlson comorbidity index, smoking-related comorbidities, alcohol use disorders, morbid smoking history (status and pack-years), education, income level, creatinine level, HbA1c level, obesity, pancreatitis, hypertension, monthly income, and urbanization level

Abbreviations: BMI: body mass index; HbA1C: glycosylated hemoglobin; OGLD: oral glucose-lowering drugs; NSAID: nonsteroidal anti-inflammatory drugs.

**Table 3:** A subgroup analysis of metformin use and lung cancer risk in patients with diabetes.

Subgroups	Pooled RR			Heterogeneity		
	*N*	RR (95% CI)	*P* value	*Q* value	*P* value	*I* ^2^ (%)
Study design						
Case-control	3	0.70 (0.47-1.03)	0.07	7.51	0.02	73
Cohort	10	0.91 (0.85-0.98)	0.008	26.33	0.002	66
Study location						
Asia	4	0.76 (0.55-1.06)	0.1	16.89	0.0007	82
North America	4	0.92 (0.71-1.19)	0.51	8.43	0.04	64
Europe	5	0.90 (0.86-0.94)	<0.0001	7.33	0.12	45
Source of case						
Population-based	7	0.88 (0.76-1.01)	0.07	18.59	0.005	68
Hospital-based	6	0.89 (0.80-0.99)	0.04	16.88	0.005	70
Control drugs						
None	8	0.89 (0.77-1.03)	0.13	22.37	0.002	69
Sulfonylurea	5	0.91 (0.86-0.96)	0.001	5.09	0.28	21
Insulin	3	0.97 (0.75-1.26)	0.84	0.06	0.97	0
Adjustment						
BMI	8	0.91 (0.80-1.03)	0.12	16.41	0.02	57
Smoking	6	0.86 (0.75-1.00)	0.05	14.11	0.01	65
HbA1C	4	0.83 (0.65-1.07)	0.16	8.31	0.04	64
Alcohol	5	0.93 (0.83-1.05)	0.27	8.07	0.09	50
Glucose-lowering drugs	4	0.90 (0.84-0.97)	0.004	3.51	0.32	15

## Data Availability

All data generated or analyzed during this study are included in this published article (and Supplementary Materials available here).

## Supplementary Materials

Supplementary Materials For the PRISMA 2009 checklist.doc, it is described as the Preferred Reporting Items for Systematic Reviews and Meta-Analyses (PRISMA) checklists of which the 27 checklist items pertain to the content of a systematic review and meta-analysis, which include the title, abstract, methods, results, discussion, and funding.
